# Seafood intake in childhood/adolescence and the risk of obesity: results from a Nationwide Cohort Study

**DOI:** 10.1186/s12937-024-00986-6

**Published:** 2024-07-16

**Authors:** Tianyue Zhang, Hao Ye, Xiaoqin Pang, Xiaohui Liu, Yepeng Hu, Yuanyou Wang, Chao Zheng, Jingjing Jiao, Xiaohong Xu

**Affiliations:** 1 Department of Endocrinology, The Second Affiliated Hospital, Zhejiang University School of Medicine, Hangzhou, Zhejiang China; 2 Department of Nutrition, School of Public Health, Zhejiang University School of Medicine, Hangzhou, Zhejiang China; 3 Department of Endocrinology, Haiyan People’s Hospital, Jiaxing, Zhejiang China

**Keywords:** Obesity, Childhood and adolescents, Seafood, China health and nutrition survey, Prospective cohort study

## Abstract

**Background & aims:**

Obesity has been linked to various detrimental health consequences. While there is established evidence of a negative correlation between seafood consumption and obesity in adults, the current research on the association between seafood intake in childhood/adolescence and the risk of obesity is lacking. Our aim was to evaluate the association between seafood intake in childhood/adolescence and the risk of obesity in a Chinese nationwide cohort.

**Methods:**

We utilized data from the China Health and Nutrition Survey (CHNS) from the year of 1997 to 2015. Seafood consumption was evaluated through 3-day 24-hour recalls. In our study, overweight/obesity status was determined based on the Chinese Criteria of Overweight and Obesity in School-age Children and Adolescents (WS/T 586–2018), while abdominal obesity status was determined according to the Chinese Criteria of Waist Circumference Screening Threshold among Children and Adolescents (WS/T 611–2018).

**Results:**

During an average follow-up of 7.9 years, 404 cases developed overweight/obesity among 2206 participants in the seafood-overweight/obesity analysis, while 381 cases developed abdominal obesity among 2256 participants in the seafood-abdominal-obesity analysis. The high-consumer group was associated with 35% lower risk of overweight/obesity risk and 26% lower risk of abdominal obesity after fully adjusting for sociodemographic and lifestyle factors, compared with the non-consumer group. Considering different cooking methods, boiled seafood consumption was associated with 43% lower risk of overweight/obesity and 23% lower risk of abdominal obesity in the fully adjusted model, while stir-fried seafood did not demonstrate a statistical significance.

**Conclusion:**

Higher intake of seafood in childhood/adolescents, particularly in a boiled way, was associated with lower obesity risk.

**Supplementary Information:**

The online version contains supplementary material available at 10.1186/s12937-024-00986-6.

## Introduction

Obesity is a significant public health issue that is currently experiencing a consistent increase risk across numerous countries. The prevalence of overweight, including obesity, among children and adolescents has risen dramatically from 8% in 1990 to 20% in 2022 [1, 2]. Obesity that occurs during critical growth and development periods can affect growth, hormonal balances, puberty and psychosocial health. Child, adolescent and adult obesity are all associated with an increased risk of morbidity, including an increased risk of chronic diseases such as type 2 diabetes, cardiovascular diseases, and certain types of cancer [3, 4]. In addition to high body mass index (BMI), abdominal obesity is also associated with multiple cardiovascular complications [5]. This places a substantial burden on healthcare systems and diminishes the quality of life for affected individuals.

Establishing healthy eating habits early in life can help individuals avoid obesity and further improve their health later in life [6]. The relationship between seafood consumption in childhood/adolescence and the risk of obesity has received significant attention. Seafood, encompassing fish, shrimp, crabs, and shellfish, is widely recognized for its high nutritional value [7]. According to the 2022 Chinese Dietary Guidelines [8], children/adolescents are recommended to consume 40–75 g of seafood per day, emphasizing the importance of including seafood in daily diets. Researches on the health benefits of consuming seafood have mainly focused on omega-3 fatty acids, with our own research as well as other clinical trials and animal studies confirming their positive effects [9–12]. Seafood also provides high-quality protein and essential nutrients like vitamin D, vitamin B12, niacin, pantothenic acid, iodine, and selenium. Additionally, seafood is frequently regarded as a more advantageous substitute for alternative protein sources, such as red and processed meats, which have been associated with an elevated susceptibility to obesity and its associated health complications [13–16].

While there are several observational and intervention research supporting the positive effects of seafood consumption on weight loss in adults, the relationship between seafood intake in childhood/adolescence and obesity in later life has yet to be definitively established [17]. Besides, assessing the link between seafood intake in childhood/adolescence and obesity faces challenges due to differences in dietary assessment methods, seafood preparation techniques, and potential confounding variables like physical activity levels, socioeconomic status, and overall dietary habits [18]. Boiling seafood is a healthy cooking method that retains its natural nutritional benefits, while frying adds unhealthy fats and calories [19, 20]. However, there are currently no studies confirming how different cooked seafood intake in childhood/adolescence and obesity affect obesity.

To full these gaps, we utilized data from the China Health and Nutrition Survey (CHNS) from the year of 1997 to 2015 and evaluated the association between seafood intake in childhood/adolescence and the risk of obesity in later life in this Chinese nationwide cohort.

## Materials & methods

### Study population

The details of the CHNS had been described elsewhere [14, 21, 22]. Briefly, it is a household-based and nationwide study initiated in 1989 and conducted every 2–3 years to evaluate the impact of societal and economic transformations on health-related outcomes and nutrition status in China. Utilizing a multistage random-cluster sampling process, samples are drawn from nine provinces and three autonomous cities added in 2011. Trained interviewers survey all members of selected households. To date, data collection has been carried out across ten waves (1989, 1991, 1993, 1997, 2000, 2004, 2006, 2009, 2011, and 2015). Since the 1989 round only included adults aged 20–45y, and the food codes from the 1991 to 1993 rounds did not correspond with the Chinese Food Composition Table (FCT), participants in the current analysis were recruited from 1997 to 2011 round. We included people participated in at least two surveys in 1997, 2000, 2004, 2006, 2009, 2011, and 2015. Follow-up time was defined as the time between the first survey and the last survey, for an average of 7.9 years. The study was approved by the institutional review committees of the University of North Carolina and the National Institute of Nutrition and Food Safety, Chinese Center for Disease Control and Prevention. All the participants provided written informed consent.

We screened 29,476 participants from the CHNS cohort and two study populations were obtained. After excluding people who were dropped-out, missed data on BMI, with cardiovascular disease (CVD)/cancer/diabetes or overweight/obese at baseline from 5020 participants aged 6–18 years with complete dietary information, 2206 people were finally included in the study on overweight/obesity (Figure S1). After excluding people who were dropped-out, missed data on waist circumference, with CVD/cancer/diabetes, or had abdominal obesity at baseline from 4691 participants aged 7–18 years with complete dietary information, 2256 people were finally included in the study on abdominal obesity (Figure S2).

### Outcome ascertainment

The height and weight of each participant in each interview were measured by well-trained staffs with the use of standard protocol and instruments. BMI was calculated by dividing body weight by the square of body height (kg/m^2^). In our study, for participants no more than 18 years old during the follow up, overweight/obesity status was established using the Chinese Criteria of Overweight and Obesity in School-age Children and Adolescents (WS/T 586–2018), whereas childhood abdominal obesity status was determined in accordance with the Chinese Criteria of Waist Circumference Screening Threshold among Children and Adolescents (WS/T 611–2018). References to the specific criteria can be found at https://www.nssi.org.cn/nssi/front/108442010.html and https://www.nssi.org.cn/nssi/front/108558216.html, respectively. For participants over 18 years old during the follow up, overweight/obesity status was determined as BMI of more than 24 kg/m^2^, and abdominal obesity standard was determined as waist circumference ≥ 88 cm for women and ≥ 102 cm for men [23].

### Dietary assessment

Dietary intake was evaluated using three consecutive 24-hour dietary recalls, a validated method for assessing dietary intake [24, 25]. Days for the recalls were randomly selected from Monday to Sunday, ensuring inclusion of at least one weekend day. Participants were instructed to adhere to their usual dietary habits, and provided detailed information on food consumption both at home and away from home to trained interviewers during the survey period. During the interview, participants were asked to provide detailed information regarding their food consumption over a 24-hour period, including the type and quantity of each item consumed, as well as the meal type and location of consumption. The estimated amount of food in each dish was determined from household inventory, and individuals reported the proportion of each dish consumed. Seafood intake included fish, shrimp, crab, shellfish, etc. and was calculated as a cumulative mean in CHNS to represent long-term diet and minimize within-person variation. Participants were categorized as seafood non-consumers, low-consumers (below the median of seafood consumption), and high-consumers (above the median of seafood consumption). The cooking method of seafood (boiled or fried) was collected by the CHNS dietary questionnaire (https://www.cpc.unc.edu/projects/china/data/questionnaires/c00nutr_c.0203.pdf). Other cooking methods (such as raw food) were not explored since the very minimal intake level. Additionally, data on soft drink, sugared-sweetened fruit drink, and alcohol consumption over the past year were collected through a food frequency questionnaire utilizing 5 categories ranging from almost daily to less than once per month. Food consumption and nutrient intake data from a variety of foods were evaluated utilizing appropriate editions of the Chinese FCT. When calculating the Alternate Healthy Eating Index (AHEI) based on AHEI-2010 [25, 26], we left trans fatty acids (was not collected in CHNS), alcohol (was already adjusted separately in the covariates), polyunsaturated fatty acid (PUFA) and n-3 PUFA (were strongly correlated with seafood intake and might lead to overadjustment) out of consideration. The AHEI in current analysis covered seven components: for vegetables, fruit, cereal fibers, nuts and legumes, higher consumption was better; for sugar-sweetened beverage (SSB), red and processed meat, and sodium, lower consumption was better. Each component is given a minimum score of 0 to indicate “worst” intake of that kind(s) of nutrient(s) and is given a maximum score of 10 to indicate “best” intake of that kind(s) of nutrient(s). Therefore, the total score ranged from 0 to 70. A higher AHEI score indicated a healthier dietary quality.

### Covariates

To control for potential confounding factors, a range of covariates collected through standardized questionnaires by professional staffs were considered in the analysis. Among the covariates, age and seafood intake in remaining cooking method were included in the analysis as continuous variables, and the other variables were included in the analysis as categorical variables, with the following classifications: income, urbanization score, energy intake, AHEI were classified according to tertiles; nationality was divided into Han, non-Han; education was divided into primary school, middle school, high school, college and above, missing; area was divided into north, east, south central, south west; physical activity was divided into very light to light, moderate, heavy to very heavy; smoking was divided into current, former, never; drinking was divided into yes, no; baseline BMI group were classified according to quartiles; baseline waist circumference was categorized as < 75th percentile and 75-90th percentile. For missing data, a missing indicator category was employed when necessary.

### Statistical analysis

Descriptive statistics were used to summarize the characteristics of the study population and the risk of obesity within the cohort. Continuous variables were presented as means with standard errors (SEs), while categorical variables were presented as frequencies and percentages. Multivariable Cox proportional hazards regression analyses were conducted to estimate the hazard ratios (HRs) and 95% confidence intervals (CIs) for obesity risk with seafood consumption. Multivariable regression models were employed to account for potential confounding factors, with model 1 adjusted for age and gender (male, female), model 2 further adjusted for nationality (Han, non-Han), income (tertiles), education (primary school, middle school, high school, college and above, missing), area (north, east, south central, south west), urbanization score (tertiles), physical activity (very light to light, moderate, heavy to very heavy), smoking (current, former, never), drinking (yes, no), baseline BMI group (quartiles, only in overweight/obesity analysis), baseline waist circumference group (only in abdominal-obesity analysis), seafood intake in remaining cooking method (in cooking-method analysis) and model 3 further adjusted for energy intake (tertiles), AHEI (tertiles). Tests for trends were assessed by calculating the median value in each frequency of seafood consumption as continuous variables. Subgroup analyses were carried out via introducing a cross-product term to examine whether the association between seafoods intake and the risk of obesity stratified by various demographic and lifestyle factors. The adjusted variables in subgroup analysis were consistent with model 3.

Sensitivity analyses were performed to further get rid of the effects of other potential risk factors on obesity. We excluded the participants aged > 18 at the end of follow-up, and participants with obesity during the initial 2 years, respectively. The covariates including insurance, and carbohydrate, fat and protein intake were further successively considered and adjusted.

Two-sided probability values < 0.05 were considered statistically significant. All analyses were conducted using the SAS statistical package (version 9.4, SAS Institute).

## Results

### Population characteristics

Baseline characteristics of participants in each group of seafood consumption in the study were shown in Table 1. In the seafood-overweight/obesity analysis, 52.5% of the participants were male, with a mean (standard deviation) age of 10.9 (3.3) years old at baseline and 18.7 (6.2) years old at the end of follow-up. In the seafood-abdominal obesity analysis, 55.1% of the participants were male, with a baseline age of 11.2 (3.1) years old and 19.0 (6.2) years old at the end of follow-up. Compared with participants in the non-consumer group and low-consumer group, participants in the high-consumer group were more likely to have an education level greater than middle school, live in urban areas, and have higher household income and urbanization index.

**Table 1:** Baseline characteristics of participants according to the consumption of total seafood in CHNS 1997–2011

Characteristics	Seafood-overweight/obesity analysis				Seafood-abdominal-obesity analysis		
	Non-consumer	Low-consumer	High-consumer		Non-consumer	Low-consumer	High-consumer
Seafood intake range (g·2000kcal^− 1^d^− 1^)	0	0 ~ 37.4	> 37.4		0	0 ~ 38.2	> 38.2
N	1047	579	580		1078	589	589
Age (years)	10.8 ± 0.1	10.9 ± 0.1	11.1 ± 0.1		11.2 ± 0.1	11.1 ± 0.1	11.3 ± 0.1
Waist circumference (cm)	59.7 ± 0.3	59.8 ± 0.4	60.1 ± 0.3		59.5 ± 0.2	59.7 ± 0.3	60.0 ± 0.3
BMI (kg/m^2^)	16.4 ± 0.1	16.4 ± 0.1	16.7 ± 0.1		16.9 ± 0.1	17.0 ± 0.1	17.3 ± 0.1
Male (%)	533 (50.9)	327 (56.5)	299 (51.6)		562 (52.1)	352 (59.8)	336 (57.1)
Han (%)	846 (80.8)	511 (88.3)	550 (94.8)		874 (81.1)	512 (86.9)	557 (94.6)
Greater than middle school (%)	33 (3.2)	24 (3.9)	52 (8.9)		31 (2.9)	21 (3.4)	51 (8.7)
North site (%)	229 (21.9)	112 (19.3)	96 (16.6)		227 (21.1)	117 (19.9)	96 (16.3)
Urban site (%)	254 (24.3)	189 (32.6)	243 (41.9)		246 (22.8)	207 (35.1)	259 (44.0)
Household income (yuan/yr)	19659.1 ± 817.2	19753.9 ± 685.7	25849.0 ± 1011.2		19137.7 ± 774.9	19155.5 ± 681.0	25962.8 ± 994.1
Urbanization index	49.5 ± 0.6	56.9 ± 0.8	62.7 ± 0.8		49.2 ± 0.6	57.8 ± 0.8	63.4 ± 0.8
Moderate-to-vigorous activity (%)	855 (81.7)	460 (79.4)	477 (81.2)		904 (83.9)	474 (80.8)	488 (82.9)
Current drinker (%)	25 (2.4)	12 (2.1)	17 (2.9)		26 (2.4)	12 (2.0)	18 (3.1)
Current smoker (%)	11 (1.1)	8 (1.4)	8 (1.4)		10 (0.9)	7 (1.2)	8 (1.4)
Energy intake (kcal/day)	1867.5 ± 16.2	2079.7 ± 28.1	1892.7 ± 19.6		1897.0 ± 15.6	2105.0 ± 27.9	1936.0 ± 20.1
AHEI score	35.6 ± 0.2	34.8 ± 0.2	34.1 ± 0.2		35.9 ± 0.2	34.9 ± 0.2	34.1 ± 0.2
Fish intake (g·2000kcal^− 1^d^− 1^)	0.0 ± 0.0	19.1 ± 0.4	66.0 ± 1.6		0.0 ± 0.0	19.7 ± 0.4	67.1 ± 1.5
Shrimp/crab intake (g·2000kcal^− 1^d^− 1^)	0.0 ± 0.0	0.8 ± 0.1	6.7 ± 0.9		0.0 ± 0.0	0.7 ± 0.1	6.4 ± 0.9
Shellfish intake (g·2000kcal^− 1^d^− 1^)	0.0 ± 0.0	0.3 ± 0.1	1.9 ± 0.4		0.0 ± 0.0	0.3 ± 0.1	1.4 ± 0.3
Boiled seafoods intake (g·2000kcal^− 1^d^− 1^)	0.0 ± 0.0	10.5 ± 0.5	41.1 ± 1.6		0.0 ± 0.0	11.1 ± 0.5	41.0 ± 1.6
Stir-fried seafoods intake (g·2000kcal^− 1^d^− 1^)	0.0 ± 0.0	6.4 ± 0.4	21.6 ± 1.4		0.0 ± 0.0	6.7 ± 0.4	23.5 ± 1.5

BMI: body mass index; AHEI: alternative healthy eating index. Values are expressed as means (SE) or n (%) unless stated otherwise.

### Seafood consumption in childhood/adolescence and overweight/obesity

404 overweight/obesity cases were identified during a total of 17,155 person-years of follow-up. The high-consumer group was associated with 22% lower of overweight/obesity risk in the age- and sex-adjusted model, compared with the non-consumer group (Table 2). The negative association was still significant after fully adjusting for sociodemographic and dietary factors (HR _High−consumer vs. Non−consumer_ 0.65; 95% CI 0.49 to 0.85; *P* for trend < 0.001). Considering different cooking methods, boiled seafood consumption was associated with 43% lower overweight/obesity risk in the fully adjusted model (*P* for trend < 0.001). However, such an association was not observed for stir-fried seafood consumption.

**Table 2:** Hazard ratios (95% CI) of seafood consumption for the risk of overweight/abdominal obesity in CHNS 1997–2011

	Dietary seafood intake (g·2000 kcal-1d-1)					
	Non-consumer	Low-consumer	High-consumer	*P*-trend		
**Seafood-overweight/obesity analysis**						
Total seafood						
Range (g·2000 kcal-1d-1)	0	0 ~ 37.4	> 37.4			
Cases/n	196/1047	110/579	98/580			
Person-years	7357	5131	4667			
Model 1	1 (Ref.)	0.77 (0.61–0.97)	0.78 (0.61–0.99)	0.023		
Model 2	1 (Ref.)	0.68 (0.53–0.87)	0.64 (0.49–0.84)	< 0.001		
Model 3	1 (Ref.)	0.70 (0.54–0.89)	0.65 (0.49–0.85)	< 0.001		
Boiled seafood						
Range (g·2000 kcal-1d-1)	0	0 ~ 28.9	> 28.9			
Cases/n	267/1410	85/398	52/398			
Person-years	10,376	3588	3191			
Model 1	1 (Ref.)	0.88 (0.69–1.13)	0.63 (0.47–0.85)	0.003		
Model 2	1 (Ref.)	0.63 (0.47–0.85)	0.57 (0.42–0.78)	< 0.001		
Model 3	1 (Ref.)	0.88 (0.68–1.14)	0.57 (0.42–0.78)	< 0.001		
Stir-fried seafood						
Range (g·2000 kcal-1d-1)	0	0 ~ 21.2	> 21.2			
Cases/n	297/1652	56/277	51/277			
Person-years	12,298	2689	2168			
Model 1	1 (Ref.)	0.86 (0.64–1.14)	1.04 (0.77–1.40)	0.847		
Model 2	1 (Ref.)	0.75 (0.55–1.01)	0.86 (0.62–1.18)	0.145		
Model 3	1 (Ref.)	0.76 (0.56–1.02)	0.86 (0.62–1.18)	0.150		
**Seafood-abdominal-obesity analysis**						
Total seafood						
Range (g·2000 kcal-1d-1)	0	0 ~ 38.2	> 38.2			
Cases/n	176/1078	108/589	97/589			
Person-years	7786	5158	4781			
Model 1	1 (Ref.)	0.95 (0.75–1.21)	0.90 (0.71–1.16)	0.418		
Model 2	1 (Ref.)	0.77 (0.59–0.99)	0.72 (0.55–0.94)	0.013		
Model 3	1 (Ref.)	0.78 (0.61–1.01)	0.74 (0.56–0.97)	0.024		
Boiled seafood						
Range (g·2000 kcal-1d-1)	0	0 ~ 28.9	> 28.9			
Cases/n	245/1440	70/408	66/408			
Person-years	10,780	3683	3262			
Model 1	1 (Ref.)	0.85 (0.65–1.11)	0.88 (0.67–1.16)	0.250		
Model 2	1 (Ref.)	0.78 (0.59–1.03)	0.74 (0.56–0.99)	0.022		
Model 3	1 (Ref.)	0.82 (0.62–1.08)	0.77 (0.58–1.03)	0.046		
Stir-fried seafood						
Range (g·2000 kcal-1d-1)	0	0 ~ 21.4	> 21.4			
Cases/n	270/1674	64/291	47/291			
Person-years	12,521	2894	2310			
Model 1	1 (Ref.)	1.05 (0.80–1.38)	1.00 (0.73–1.36)	0.920		
Model 2	1 (Ref.)	0.88 (0.66–1.17)	0.87 (0.63–1.20)	0.304		
Model 3	1 (Ref.)	0.90 (0.67–1.20)	0.88 (0.64–1.22)	0.361		

Model 1 adjusted for age and gender (male, female). Model 2 further adjusted for nationality (Han, non-Han), income (tertiles), education (primary school, middle school, high school, college and above, missing), area (north, east, south central, south west), urbanization score (tertiles), physical activity (very light to light, moderate, heavy to very heavy), smoking (current, former, never), drinking (yes, no), baseline BMI group (quartiles, only in overweight/obesity analysis), baseline waist circumference group (only in abdominal-obesity analysis), seafood intake in remaining cooking method (in cooking-method analysis). Model 3 further adjusted for energy intake (tertiles), AHEI (tertiles)

### Seafood consumption in childhood/adolescence and abdominal obesity

381 cases with abdominal obesity were identified during a total of 17,725 person-years of follow-up. The association was not observed in the age- and sex-adjusted model. However, after adjusting for multivariate factors, including dietary factors, compared with the lowest quartile, those in the high-consumer group had 26% lower risk (HR _High−consumer vs. Non−consumer_ 0.74; 95% CI 0.56 to 0.97; *P* for trend = 0.024). Given various cooking methods, boiled seafood intake showed a negative association with abdominal obese risk (HR _High−consumer vs. Non−consumer_: 0.77; 95% CI 0.58 to 1.03; *P* for trend = 0.046) in the multivariate-adjusted model whereas stir-fried seafood intake had no significant association (Table 2).

### Sensitivity analyses

In sensitivity analyses (Table 3), the inverse association between total seafood intake and boiled seafood intake, but not stir-fried seafood intake, with obesity has been mostly observed after excluding participants aged > 18 when obesity occurred or at the end of the follow-up, individuals who had obesity during the initial two years, and after adjusting for insurance coverage, carbohydrate, fat and protein intake. However, after excluding participants aged > 18 years at the end, the significance of total seafood intake or boiled seafood intake with abdominal obesity disappeared.

**Table 3:** Sensitivity analysis of seafood consumption for the risk of overweight/abdominal obesity in CHNS 1997–2011

	Cases/*n*	Dietary seafood intake (g·2000kcal^− 1^d^− 1^)			
		Non-consumer	Low-consumer	High-consumer	*P* for trend
**Seafood-overweight/obesity analysis**					
Excluding participants aged > 18 at the end of the follow-up					
Total seafood	171/2137	1 (Ref.)	0.61 (0.41–0.92)	0.60 (0.39–0.91)	0.011
Boiled seafood	171/2137	1 (Ref.)	0.95 (0.64–1.42)	0.56 (0.34–0.93)	0.037
Stir-fried seafood	171/2137	1 (Ref.)	0.85 (0.53–1.39)	0.90 (0.55–1.48)	0.562
Excluding cases of overweight/obesity within the first two years					
Total seafood	391/2193	1 (Ref.)	0.68 (0.52–0.88)	0.65 (0.49–0.85)	0.001
Boiled seafood	391/2193	1 (Ref.)	0.87 (0.67–1.14)	0.58 (0.42–0.79)	< 0.001
Stir-fried seafood	391/2193	1 (Ref.)	0.78 (0.58–1.06)	0.86 (0.62–1.18)	0.174
Adjusted for insurance					
Total seafood	404/2206	1 (Ref.)	0.70 (0.54–0.90)	0.64 (0.49–0.84)	< 0.001
Boiled seafood	404/2206	1 (Ref.)	0.90 (0.69–1.17)	0.58 (0.42–0.79)	0.001
Stir-fried seafood	404/2206	1 (Ref.)	0.75 (0.56–1.02)	0.85 (0.62–1.17)	0.140
*Adjusted for carbohydrate, fat and protein intake					
Total seafood	404/2206	1 (Ref.)	0.68 (0.53–0.88)	0.63 (0.48–0.84)	< 0.001
Boiled seafood	404/2206	1 (Ref.)	0.86 (0.66–1.12)	0.57 (0.42–0.79)	< 0.001
Stir-fried seafood	404/2206	1 (Ref.)	0.73 (0.54-1.00)	0.83 (0.60–1.15)	0.114
**Seafood-abdominal-obesity analysis**					
Excluding participants aged > 18 at the end of the follow-up					
Total seafood	242/1240	1 (Ref.)	0.79 (0.57–1.11)	0.84 (0.59–1.20)	0.303
Boiled seafood	242/1240	1 (Ref.)	0.76 (0.53–1.09)	0.81 (0.57–1.17)	0.170
Stir-fried seafood	242/1240	1 (Ref.)	1.30 (0.87–1.93)	1.13 (0.75–1.70)	0.360
Excluding cases of abdominal obesity within the first two years					
Total seafood	363/2238	1 (Ref.)	0.77 (0.59–1.01)	0.73 (0.55–0.97)	0.022
Boiled seafood	363/2238	1 (Ref.)	0.82 (0.62–1.10)	0.76 (0.57–1.03)	0.054
Stir-fried seafood	363/2238	1 (Ref.)	0.95 (0.71–1.28)	0.90 (0.64–1.25)	0.505
Adjusted for insurance					
Total seafood	381/2256	1 (Ref.)	0.78 (0.61–1.01)	0.73 (0.56–0.97)	0.022
Boiled seafood	381/2256	1 (Ref.)	0.81 (0.61–1.06)	0.76 (0.57–1.01)	0.039
Stir-fried seafood	381/2256	1 (Ref.)	0.89 (0.67–1.20)	0.88 (0.64–1.22)	0.361
*Adjusted for carbohydrate, fat and protein intake					
Total seafood	381/2256	1 (Ref.)	0.78 (0.60–1.01)	0.71 (0.53–0.95)	0.018
Boiled seafood	381/2256	1 (Ref.)	0.80 (0.60–1.06)	0.75 (0.56–1.01)	0.040
Stir-fried seafood	381/2256	1 (Ref.)	0.87 (0.64–1.17)	0.85 (0.61–1.19)	0.268

All models adjusting for covariates listed the in Model 3 of Table 2 footnote, except * indicated unadjusted for AHEI.

### Subgroup analyses

In subgroup analyses (Tables 4 and 5), compared with males, the negative relationship between boiled seafood consumption and abdominal obesity risk were stronger in females (*P* for interaction = 0.030). No significant effect of age, gender, total energy intake, AHEI index, physical activity, smoking, area, urban site, urbanization index, income, and education on the relationship between total seafood intake and obesity were noted.

**Table 4:** Subgroup analysis of total seafood consumption for the risk of overweight/abdominal obesity in CHNS 1997–2011

	Cases/*n*	Dietary seafood intake (g·2000kcal^− 1^d^− 1^)				*P* for interaction
		Non-consumer	Low-consumer	High-consumer	*P* for trend	
**Seafood-overweight/obesity analysis**						
Age (years)						0.923
6 ~ 10	209/1102	1 (Ref.)	0.53 (0.37–0.77)	0.60 (0.41–0.86)	0.004	
11 ~ 13	92/586	1 (Ref.)	1.24 (0.73–2.13)	0.71 (0.38–1.31)	0.346	
14 ~ 18	103/518	1 (Ref.)	0.73 (0.42–1.25)	0.62 (0.33–1.17)	0.121	
Gender						0.207
Men	292/1159	1 (Ref.)	0.74 (0.55-1.00)	0.67 (0.48–0.92)	0.011	
Women	112/1047	1 (Ref.)	0.51 (0.31–0.85)	0.62 (0.37–1.03)	0.044	
Nationality						0.294
Han	370/1907	1 (Ref.)	0.76 (0.27–2.12)	0.69 (0.14–3.43)	0.547	
Non-Han	34/299	1 (Ref.)	0.67 (0.52–0.88)	0.64 (0.49–0.85)	0.001	
Physical activity						0.176
Very light to moderate	335/1851	1 (Ref.)	0.73 (0.55–0.97)	0.59 (0.44–0.79)	< 0.001	
Heavy to very heavy	69/355	1 (Ref.)	0.90 (0.67–1.21)	0.55 (0.39–0.77)	0.887	
Urbanization index						0.851
< Median	215/1106	1 (Ref.)	0.88 (0.62–1.24)	0.58 (0.39–0.87)	0.010	
≥Median	189/1100	1 (Ref.)	0.57 (0.39–0.84)	0.63 (0.43–0.93)	0.027	
Income						0.821
< Median	196/1091	1 (Ref.)	0.70 (0.49-1.00)	0.63 (0.42–0.96)	0.018	
≥Median	204/1090	1 (Ref.)	0.73 (0.50–1.07)	0.68 (0.47–0.98)	0.042	
Smoking						0.283
Nonsmoker	292/1611	1 (Ref.)	0.69 (0.51–0.93)	0.72 (0.52–0.98)	0.028	
Former/current smoker	112/595	1 (Ref.)	0.64 (0.38–1.07)	0.48 (0.28–0.85)	0.008	
Area						0.784
North	88/437	1 (Ref.)	0.74 (0.41–1.35)	1.29 (0.71–2.36)	0.654	
East	71/355	1 (Ref.)	0.39 (0.20–0.73)	0.36 (0.19–0.68)	0.002	
South central	195/1030	1 (Ref.)	0.72 (0.50–1.05)	0.68 (0.46–0.99)	0.044	
South west	50/384	1 (Ref.)	0.71 (0.27–1.87)	1.58 (0.42–5.96)	0.929	
Urban site						0.554
Yes	102/686	1 (Ref.)	0.61 (0.35–1.03)	0.56 (0.33–0.96)	0.040	
No	302/1520	1 (Ref.)	0.75 (0.56–0.99)	0.69 (0.51–0.94)	0.010	
Educational level						0.605
< Middle school	340/1871	1 (Ref.)	0.65 (0.49–0.85)	0.62 (0.46–0.84)	< 0.001	
≥ Middle school	64/335	1 (Ref.)	0.96 (0.47–1.93)	0.50 (0.25–1.01)	0.053	
Total energy intake						0.449
< Median	175/1103	1 (Ref.)	0.40 (0.26–0.64)	0.75 (0.52–1.09)	0.080	
≥Median	229/1103	1 (Ref.)	0.94 (0.68–1.30)	0.59 (0.40–0.89)	0.016	
AHEI score						0.255
< Median	196/1103	1 (Ref.)	0.63 (0.43–0.93)	0.55 (0.37–0.82)	0.003	
≥Median	208/1103	1 (Ref.)	0.75 (0.52–1.07)	0.75 (0.51–1.10)	0.111	
**Seafood-abdominal-obesity analysis**						
Age (years)						0.119
7 ~ 10	189/1093	1 (Ref.)	0.72 (0.50–1.06)	0.91 (0.62–1.33)	0.544	
11 ~ 13	111/619	1 (Ref.)	0.90 (0.55–1.46)	0.62 (0.37–1.05)	0.078	
14 ~ 18	81/544	1 (Ref.)	0.78 (0.45–1.38)	0.44 (0.22–0.88)	0.022	
Gender						0.075
Men	223/1250	1 (Ref.)	0.66 (0.47–0.93)	0.87 (0.61–1.23)	0.361	
Women	158/1006	1 (Ref.)	1.03 (0.70–1.52)	0.59 (0.37–0.94)	0.038	
Nationality						0.912
Han	343/1943	1 (Ref.)	0.78 (0.60–1.03)	0.75 (0.57-1.00)	0.045	
Non-Han	38/313	1 (Ref.)	0.59 (0.24–1.48)	0.54 (0.14–2.12)	0.246	
Physical activity						0.626
Very light to moderate	331/1940	1 (Ref.)	0.81 (0.61–1.06)	0.74 (0.55–0.99)	0.037	
Heavy to very heavy	50/316	1 (Ref.)	0.64 (0.30–1.36)	0.76 (0.32–1.79)	0.424	
Urbanization index						0.084
< Median	187/1121	1 (Ref.)	0.63 (0.43–0.92)	0.45 (0.29–0.72)	< 0.001	
≥Median	194/1135	1 (Ref.)	0.96 (0.65–1.41)	1.07 (0.72–1.57)	0.716	
Income						0.431
< Median	195/1115	1 (Ref.)	0.60 (0.42–0.86)	0.56 (0.36–0.85)	0.002	
≥Median	182/1113	1 (Ref.)	0.97 (0.65–1.43)	0.96 (0.65–1.42)	0.827	
Smoking						0.855
Nonsmoker	280/1630	1 (Ref.)	0.73 (0.54–0.98)	0.75 (0.54–1.03)	0.059	
Former/current smoker	101/626	1 (Ref.)	1.04 (0.63–1.74)	0.74 (0.43–1.28)	0.305	
Area						0.329
North	81/440	1 (Ref.)	1.13 (0.65–1.97)	1.35 (0.76–2.42)	0.312	
East	110/445	1 (Ref.)	0.71 (0.44–1.14)	0.71 (0.42–1.19)	0.151	
South central	157/997	1 (Ref.)	0.75 (0.49–1.13)	0.63 (0.40–0.97)	0.036	
South west	33/374	1 (Ref.)	0.46 (0.13–1.72)	1.39 (0.26–7.39)	0.661	
Urban site						0.704
Yes	114/712	1 (Ref.)	0.77 (0.45–1.29)	0.67 (0.39–1.15)	0.161	
No	267/1544	1 (Ref.)	0.75 (0.55–1.02)	0.80 (0.57–1.12)	0.113	
Educational level						0.852
< Middle school	333/1947	1 (Ref.)	0.72 (0.54–0.95)	0.75 (0.56-1.00)	0.040	
≥ Middle school	48/309	1 (Ref.)	1.21 (0.57–2.59)	0.46 (0.19–1.12)	0.101	
Total energy intake						0.658
< Median	189/1128	1 (Ref.)	0.86 (0.59–1.24)	0.73 (0.50–1.08)	0.110	
≥Median	192/1128	1 (Ref.)	0.74 (0.52–1.07)	0.77 (0.52–1.15)	0.170	
AHEI score						0.559
< Median	185/1128	1 (Ref.)	0.99 (0.67–1.45)	0.88 (0.59–1.31)	0.525	
≥Median	196/1128	1 (Ref.)	0.69 (0.48–0.99)	0.72 (0.48–1.07)	0.063	

All models adjusting for covariates listed the in Model 3 of Table 2 footnote.

**Table 5:** Subgroup analysis of boiled seafood consumption for the risk of overweight/abdominal obesity in CHNS 1997–2011

	Cases/*n*	Boiled seafood intake (g·2000kcal^− 1^d^− 1^)				*P* for interaction
		Non-consumer	Low-consumer	High-consumer	*P* for trend	
**Seafood-overweight/obesity analysis**						
Age (years)						0.999
6 ~ 10	209/1102	1 (Ref.)	0.80 (0.56–1.15)	0.46 (0.29–0.71)	< 0.001	
11 ~ 13	92/586	1 (Ref.)	1.27 (0.70–2.30)	1.06 (0.58–1.92)	0.743	
14 ~ 18	103/518	1 (Ref.)	0.87 (0.50–1.51)	0.51 (0.23–1.14)	0.116	
Gender						0.124
Men	292/1159	1 (Ref.)	0.98 (0.72–1.34)	0.66 (0.46–0.95)	0.043	
Women	112/1047	1 (Ref.)	0.68 (0.40–1.15)	0.49 (0.25–0.94)	0.018	
Nationality						0.154
Han	370/1907	1 (Ref.)	2.72 (0.74–10.03)	0.72 (0.16–3.14)	0.922	
Non-Han	34/299	1 (Ref.)	0.87 (0.66–1.15)	0.57 (0.41–0.79)	< 0.001	
BMI						0.677
< Median	138/1103	1 (Ref.)	0.82 (0.52–1.29)	0.71 (0.42–1.19)	0.160	
≥Median	266/1103	1 (Ref.)	0.91 (0.66–1.27)	0.54 (0.36–0.80)	0.003	
Physical activity						0.392
Very light to moderate	335/1851	1 (Ref.)	0.90 (0.67–1.21)	0.55 (0.39–0.77)	< 0.001	
Heavy to very heavy	69/355	1 (Ref.)	0.63 (0.32–1.24)	0.98 (0.40–2.42)	0.510	
Urbanization index						0.669
< Median	215/1106	1 (Ref.)	0.87 (0.59–1.28)	0.47 (0.28–0.78)	0.004	
≥Median	189/1100	1 (Ref.)	0.92 (0.63–1.34)	0.63 (0.41–0.96)	0.037	
Income						0.995
< Median	196/1091	1 (Ref.)	0.79 (0.53–1.17)	0.54 (0.33–0.89)	0.012	
≥Median	204/1090	1 (Ref.)	1.13 (0.77–1.64)	0.64 (0.42–0.97)	0.069	
Smoking						0.336
Nonsmoker	292/1611	1 (Ref.)	0.89 (0.66–1.20)	0.64 (0.45–0.91)	0.015	
Former/current smoker	112/595	1 (Ref.)	0.91 (0.53–1.56)	0.44 (0.22–0.90)	0.033	
Area						0.533
North	88/437	1 (Ref.)	1.08 (0.57–2.07)	1.34 (0.69–2.60)	0.392	
East	71/355	1 (Ref.)	0.45 (0.21-1.00)	0.30 (0.15–0.61)	< 0.001	
South central	195/1030	1 (Ref.)	0.92 (0.64–1.32)	0.54 (0.34–0.85)	0.011	
South west	50/384	1 (Ref.)	1.61 (0.50–5.13)	1.24 (0.33–4.62)	0.528	
Urban site						0.321
Yes	102/686	1 (Ref.)	0.87 (0.53–1.43)	0.77 (0.43–1.37)	0.360	
No	302/1520	1 (Ref.)	0.94 (0.69–1.29)	0.48 (0.32–0.70)	< 0.001	
Educational level						0.804
< Middle school	340/1871	1 (Ref.)	0.83 (0.62–1.10)	0.57 (0.40–0.80)	0.001	
≥ Middle school	64/335	1 (Ref.)	1.14 (0.56–2.32)	0.44 (0.19–0.99)	0.077	
Total energy intake						0.059
< Median	175/1103	1 (Ref.)	0.70 (0.45–1.11)	0.95 (0.62–1.47)	0.527	
≥Median	229/1103	1 (Ref.)	1.01 (0.72–1.41)	0.39 (0.24–0.62)	< 0.001	
AHEI score						0.843
< Median	196/1103	1 (Ref.)	0.90 (0.60–1.34)	0.56 (0.35–0.89)	0.018	
≥Median	208/1103	1 (Ref.)	0.86 (0.59–1.25)	0.58 (0.37–0.90)	0.015	
**Seafood-abdominal-obesity analysis**						
Age (years)						0.793
7 ~ 10	189/1093	1 (Ref.)	0.87 (0.59–1.30)	0.76 (0.50–1.14)	0.170	
11 ~ 13	111/619	1 (Ref.)	0.70 (0.39–1.27)	0.90 (0.54–1.49)	0.539	
14 ~ 18	81/544	1 (Ref.)	0.79 (0.43–1.45)	0.54 (0.26–1.13)	0.096	
Gender						0.030
Men	223/1250	1 (Ref.)	0.78 (0.54–1.12)	0.98 (0.68–1.41)	0.669	
Women	158/1006	1 (Ref.)	0.86 (0.55–1.34)	0.57 (0.35–0.93)	0.025	
Nationality						0.671
Han	343/1943	1 (Ref.)	0.83 (0.62–1.12)	0.77 (0.57–1.04)	0.065	
Non-Han	38/313	1 (Ref.)	0.48 (0.14–1.70)	0.98 (0.31–3.11)	0.682	
Physical activity						0.375
Very light to moderate	331/1940	1 (Ref.)	0.86 (0.64–1.16)	0.78 (0.58–1.06)	0.098	
Heavy to very heavy	50/316	1 (Ref.)	0.54 (0.19–1.51)	0.77 (0.27–2.25)	0.401	
Urbanization index						0.397
< Median	187/1121	1 (Ref.)	0.75 (0.47–1.17)	0.54 (0.32–0.90)	0.011	
≥Median	194/1135	1 (Ref.)	0.90 (0.62–1.31)	0.97 (0.66–1.40)	0.793	
Income						0.844
< Median	195/1115	1 (Ref.)	0.70 (0.46–1.07)	0.65 (0.42–1.02)	0.030	
≥Median	182/1113	1 (Ref.)	0.86 (0.57–1.29)	0.90 (0.61–1.34)	0.539	
Smoking						0.979
Nonsmoker	280/1630	1 (Ref.)	0.83 (0.60–1.14)	0.76 (0.54–1.06)	0.083	
Former/current smoker	101/626	1 (Ref.)	0.76 (0.42–1.39)	0.76 (0.43–1.34)	0.284	
Area						0.282
North	81/440	1 (Ref.)	1.34 (0.71–2.52)	1.40 (0.76–2.59)	0.214	
East	110/445	1 (Ref.)	0.78 (0.43–1.41)	0.65 (0.37–1.12)	0.105	
South central	157/997	1 (Ref.)	0.84 (0.56–1.27)	0.79 (0.50–1.24)	0.277	
South west	33/374	1 (Ref.)	0.65 (0.14–3.06)	0.77 (0.09–6.36)	0.614	
Urban site						0.922
Yes	114/712	1 (Ref.)	0.82 (0.50–1.35)	0.90 (0.54–1.50)	0.652	
No	267/1544	1 (Ref.)	0.86 (0.60–1.22)	0.75 (0.52–1.09)	0.107	
Educational level						0.350
< Middle school	333/1947	1 (Ref.)	0.78 (0.58–1.05)	0.73 (0.53-1.00)	0.029	
≥ Middle school	48/309	1 (Ref.)	0.79 (0.32–1.94)	0.85 (0.37–1.97)	0.669	
Total energy intake						0.915
< Median	189/1128	1 (Ref.)	0.79 (0.52–1.20)	0.69 (0.45–1.04)	0.059	
≥Median	192/1128	1 (Ref.)	0.84 (0.57–1.23)	0.87 (0.58–1.31)	0.419	
AHEI score						0.693
< Median	185/1128	1 (Ref.)	0.68 (0.45–1.03)	0.80 (0.54–1.20)	0.196	
≥Median	196/1128	1 (Ref.)	0.97 (0.66–1.44)	0.77 (0.50–1.18)	0.260	

All models adjusting for covariates listed the in Model 3 of Table 2 footnote.

## Discussion

To our knowledge, the current 7.9-years prospective study is the first report to longitudinally assess the associations of seafood intake in childhood/adolescence and obesity in a Chinese population. In this nationwide study, it was observed that the total seafood intake and boiled seafood intake were inversely associated with both overweight/obesity and abdominal obesity. However, no such association was found in relation to stir-fried seafood intake.

Dietary habit is one of the key modifiable factors to protect health. Among the diverse kinds of foods, seafoods as vital sources of marine n-3 PUFAs, vitamin D and calcium, play a critical role in promoting health of children/adolescents, featuring protein dense and having little or no sugar or saturated fats [26]. Evidence shows that children who consumed 2 fish meals per week including one of fatty fish were less likely to show emotional and behavioral problems than those who did not [27]. A meta-analysis of 13 studies with 1,132 participants observed that fish oil (rich in marine n-3 PUFAs) intervention has a beneficial effect on insulin sensitivity in children [28]. However, in comparison to the recommended seafood intake outlined in the 2022 Chinese Dietary Guidelines [8], the participants in this study exhibited a significant deficiency in their consumption of seafood. The median daily intake of seafood in the seafood-overweight/obesity analysis was 5.36 g, with a compliance rate of only 17.63% based on the recommended intake of 50 g per day. Similarly, in the seafood-abdominal-obesity analysis, the median seafood intake was 6.14 g per day, with a compliance rate of only 18.67%. As a result, Chinese children/adolescents should be encouraged to consume more seafood in their diets.

Obesity is a growing concern worldwide, with implications for long-term health and well-being. In this context, dietary factors play a crucial role in shaping health outcomes. Seafood has been found to be related to adult weight and cardiovascular mortality [17, 29]. A meta-analysis of 17 RCTs showed more pronounced decreases of waist circumference and BMI in adults who received fish or fish oil interventions compared with the control groups [30]. However, the evidences on seafood in childhood/adolescence and obesity are still controversial. A small sample study of female adolescents found that greater fish intake corresponded to smaller changes in waist circumference [31]. Similarly, a meta-analysis including 1,028 participants from 12 RCTs observed that supplementation with fish oil could significantly reduce BMI in overweight or obese children and adolescents [32]. On the other hand, descriptive studies from Germany and South Asia showed that higher intake of fish was associated with greater BMI [33, 34]. The disparity could be due to confounding variables or variations in dietary habits and cooking methods among the different populations. For example, in Western countries, seafoods are mostly cooked in a fried way, while mostly boiled or braised in China. Altogether, our results support that higher seafood consumption in childhood/adolescence is associated with a decreased risk of obesity. Seafood is rich in marine omega-3 PUFA along with various bioactive compounds including vitamin D, selenium, iodine, taurine, and retinol. However, it is important to note that seafood may also contain harmful contaminants such as methylmercury, dioxins, biphenyl, which have been associated with promoting obesity [35]. Our research serves as a pertinent reminder that the potential protective benefits of consuming seafood in relation to obesity may outweigh the associated risks.

There is currently little literature examining different seafood cooking methods and obesity. Our findings indicated an inverse association between total seafood intake and boiled seafood intake with both overweight/obesity and abdominal obesity. This implies that a higher consumption of seafood in childhood/adolescence, particularly when prepared through boiling methods other than stir-frying, is linked to a reduced risk of these health issues. This observation aligns with existing evidence that highlights the nutritional benefits of seafood, including its omega-3 fatty acids and lean protein content, which may contribute to a healthier body composition [36–38]. Our study suggested that stir-frying, a common method of preparing seafood in various cuisines, might not confer the same protective benefits against obesity as boiled seafood. Boiled seafood, rich in protein and omega-3 fatty acids, has been shown to prevent obesity, insulin resistance and type 2 diabetes mellitus [39, 40]. In contrast, fried seafood may produce trans-fatty acids and advanced glycation end products and increase energy-density, which could counteract the possible beneficial effects of other components in seafood [20, 41–43]. This may partially explain why seafood intake did not improve cardiovascular-related mortality in a large study [29]. A previous study reported no inverse association of fried fish with mortality, which was consistent with our research results [44].

One notable finding in the study is the gender-specific correlation observed, with a particularly strong negative relationship between seafood consumption and abdominal obesity noted among girls. The exact mechanism of the gender difference has not yet been elucidated. There are several potential hypotheses. Evidence suggests that seafood consumption in boys may be accompanied by high caloric intake and/or unhealthy dietary habits, thereby diminishing the protective health effects of seafood [45]. Different genders may metabolize and respond differently to specific ingredients in seafood, which may affect the anti-obesity effect of seafood [46]. In addition, sex hormones have an important impact on the distribution and accumulation of fat tissue. Components in seafood, including omega-3 fatty acids, may have a positive effect on regulating hormone levels and improving metabolism, resulting in stronger anti-obesity effects in girls [47]. It should be noted that more scientific research and empirical data are needed to draw firm conclusions.

After excluding participants aged > 18 years at the end, the significance of total seafood intake or boiled seafood intake with abdominal obesity disappeared. This suggested that the effect of seafood in preventing abdominal obesity is more obvious in participants in late adolescence. There were several possible reasons. Firstly, the high metabolism, coupled with the growth spurts that occur during late adolescence, may make their bodies more responsive to the nutrients found in seafood, such as omega-3 fatty acids, which are known to help reduce inflammation and fat accumulation [48]. Secondly, late adolescence involves significant hormonal changes that affect body composition. Hormones like growth hormone and sex hormones (estrogen and testosterone) play crucial roles in fat distribution. The nutrients in seafood might better support hormonal balance and healthy fat distribution during this critical period [49]. Thirdly, consistent consumption of seafood can lead to a cumulative positive effect on health. By the time adolescents approach 18, the long-term benefits of seafood’s nutrients, like omega-3 fatty acids, proteins, vitamins, and minerals, may become more apparent in reducing abdominal fat [50]. Lastly, the remaining sample might not be large enough to show a statistically significant effect.

Compared to overweight/obesity defined by BMI, abdominal obesity is more likely to be ignored. However, abdominal obesity is more likely to cause unhealthy consequences such as metabolic syndrome [51]. Besides, it’s more difficult to target abdominal fat compared with weight control [52]. Our study suggested that total and boiled seafood consumption had a protective effect on both overweight/obesity and abdominal obesity, and the protective effect on abdominal obesity was particularly significant in girls or participants in late adolescence.

The strengths of this study included the prospective design, a long duration of follow-up, and detailed information on potential confounders. This study did have several limitations. First, it is important to acknowledge the potential for reverse causation to introduce bias into our results. However, in sensitivity analysis, we mitigated this concern by excluding participants who developed obesity within the first two years, ultimately finding no significant alterations to our findings. Secondly, despite our efforts to control for dietary patterns using AHEI, the complexities of interactions between nutrients and dietary patterns remain beyond the scope of our study. Thirdly, the generalizability of our findings may be limited by variations in long-term dietary habits across different populations and countries. Finally, we cannot prove a causal association in our study due to its observational nature, and there may still be residual confounding despite controlling for most potential risk factors. Future studies, including mechanistic studies and randomized controlled trials, are suggested to further explore the relationship between seafood consumption in childhood/adolescence and obesity in later life.

In summary, the comprehensive national study offers significant insights into the intricate correlation between seafood consumption in childhood/adolescence and obesity in later life, suggesting a potential protective effect of boiled seafood, especially among girls. These findings contribute to the ongoing discussion on approaches to combatting obesity and set the foundation for targeted and effective public health interventions.

### Electronic supplementary material

Below is the link to the electronic supplementary material.

Supplementary Material 1: Figure S1. Flow chart of enrolled participants in the seafood-overweight/obesity analysis. BMI: Body Mass Index

Supplementary Material 2: Figure S2. Flow chart of enrolled participants in the seafood-abdominal obesity analysis. BMI: Body Mass Index

## Data Availability

No datasets were generated or analysed during the current study.
