# Supplementary figures and images for: Seafood intake in childhood/adolescence and the risk of obesity: results from a Nationwide Cohort Study
Source: Nutr J. 2024 Jul 16;23:77. doi: 10.1186/s12937-024-00986-6 (PMC11251353; doi:10.1186/s12937-024-00986-6)

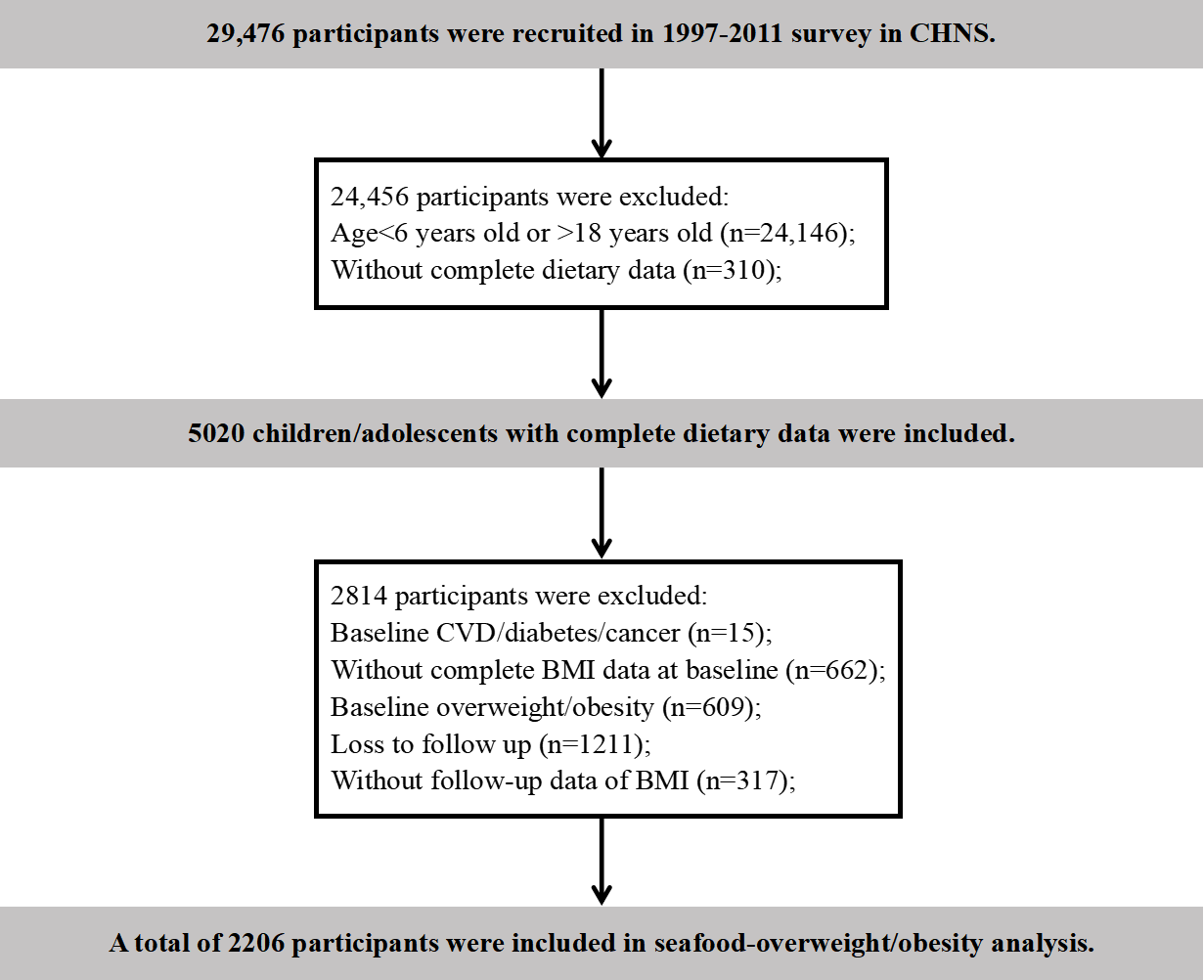

Supplement: Supplementary file 1 — Supplementary Material 1: Figure S1. Flow chart of enrolled participants in the seafood-overweight/obesity analysis. BMI: Body Mass Index [file 12937_2024_986_MOESM1_ESM.tif]

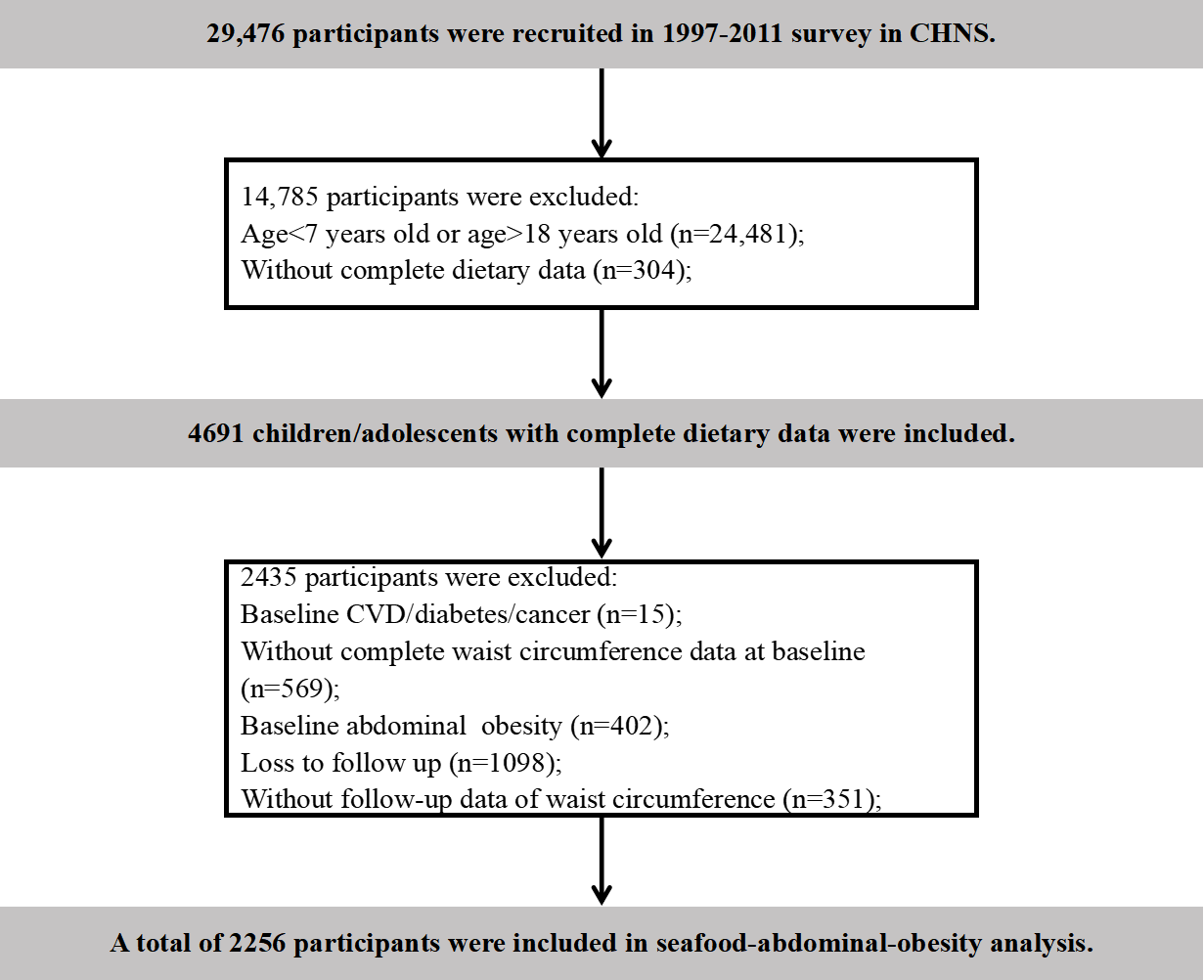

Supplement: Supplementary file 2 — Supplementary Material 2: Figure S2. Flow chart of enrolled participants in the seafood-abdominal obesity analysis. BMI: Body Mass Index [file 12937_2024_986_MOESM2_ESM.tif]
